# The mammalian efferent vestibular system utilizes cholinergic mechanisms to excite primary vestibular afferents

**DOI:** 10.1038/s41598-020-80367-1

**Published:** 2021-01-13

**Authors:** Glenn T. Schneider, Choongheon Lee, Anjali K. Sinha, Paivi M. Jordan, Joseph C. Holt

**Affiliations:** 1 Department of Otolaryngology, University of Rochester, 601 Elmwood Avenue, Box 603, Rochester, NY 14642 USA; 2 Department of Neuroscience, University of Rochester, Rochester, NY 14642 USA; 3 Department of Pharmacology and Physiology, University of Rochester, Rochester, NY 14642 USA

**Keywords:** Hair cell, Neurotransmitters, Cellular neuroscience

## Abstract

Electrical stimulation of the mammalian efferent vestibular system (EVS) predominantly excites primary vestibular afferents along two distinct time scales. Although roles for acetylcholine (ACh) have been demonstrated in other vertebrates, synaptic mechanisms underlying mammalian EVS actions are not well-characterized. To determine if activation of ACh receptors account for efferent-mediated afferent excitation in mammals, we recorded afferent activity from the superior vestibular nerve of anesthetized C57BL/6 mice while stimulating EVS neurons in the brainstem, before and after administration of cholinergic antagonists. Using a normalized coefficient of variation (CV*), we broadly classified vestibular afferents as regularly- (CV* < 0.1) or irregularly-discharging (CV* > 0.1) and characterized their responses to midline or ipsilateral EVS stimulation. Afferent responses to efferent stimulation were predominantly excitatory, grew in amplitude with increasing CV*, and consisted of fast and slow components that could be identified by differences in rise time and post-stimulus duration. Both efferent-mediated excitatory components were larger in irregular afferents with ipsilateral EVS stimulation. Our pharmacological data show, for the first time in mammals, that muscarinic AChR antagonists block efferent-mediated slow excitation whereas the nicotinic AChR antagonist DHβE selectively blocks efferent-mediated fast excitation, while leaving the efferent-mediated slow component intact. These data confirm that mammalian EVS actions are predominantly cholinergic.

## Introduction

The mammalian efferent vestibular system (EVS) originates as bilateral nuclei of predominantly cholinergic/peptidergic neurons in the dorsal brainstem^1–5^. Axons of contralateral efferent neurons cross the midline and join ipsilateral efferent neurons to exit cranial nerve VIII and innervate the vestibular labyrinth^1–4,6^. In each end organ, contralateral and ipsilateral EVS neurons extensively arborize with terminal fields that either overlap or preferentially innervate distinct neuroepithelial zones^7,8^. Within vestibular neuroepithelia, EVS neurons generate numerous synaptic varicosities abutting type II hair cells, calyx afferents, and bouton afferents^9–11^. Combined activation of synaptic mechanisms on these cellular targets produce the diverse afferent responses observed with EVS stimulation. Since the vestibular system works as a push–pull system, differential modulation of distinct synaptic mechanisms by ipsilateral and contralateral EVS neurons may be critical to balancing afferent input from both sides^8^.

While efferent-mediated afferent inhibition has been reported, the predominant response of mammalian vestibular afferents to EVS stimulation is an excitation whose amplitude and kinetics vary with afferent discharge regularity^2,12,13^. Regularly-discharging afferents were dominated by an efferent-mediated slow excitation with activation time constants measured in seconds and response durations exceeding tens of seconds. Irregularly-discharging afferents, in addition to a larger slow excitation, exhibited a prominent efferent-mediated fast excitation that peaked and decayed within 100–300 ms of both stimulus onset and termination^2,12,13^. Efferent-mediated inhibition and the two forms of efferent-mediated excitation suggest roles for multiple EVS synaptic mechanisms in mammals.

Acetylcholine (ACh) is the predominant EVS neurotransmitter in vertebrates^5,14,15^, but other neurotransmitters, including CGRP and GABA, have also been implicated. Whether release of ACh or other neurotransmitters from efferent terminals accounts for distinct afferent responses to EVS stimulation in mammals remains unresolved. Some insight is provided by EVS studies in turtle. Here, efferent-mediated fast and slow afferent excitation require activation of alpha4/alpha6/beta2-containing nicotinic ACh receptors (α4α6β2*nAChRs) and muscarinic ACh receptors (mAChRs) on afferent endings, respectively. Efferent-mediated inhibition of afferent firing, however, utilizes the sequential activation of α9nAChRs and small-conductance, calcium-activated, potassium channels (SK) in type II hair cells^16–18^. Given mammalian EVS neurons innervate the same peripheral cellular targets as turtles suggests similar synaptic mechanisms are involved.

In situ hybridization and RT-PCR data exists for numerous mAChRs and nAChRs in the mammalian vestibular periphery^19–25^. Furthermore, recent pharmacological data have confirmed that locally-released ACh activates an α9nAChR/SK mechanism in mouse type II hair cells^5,26,27^. While exogenous application of cholinergic and GABAergic agonists excites mouse vestibular afferent neurons^5,28–30^, it is unclear how these effects are related to the afferent excitation seen with direct efferent stimulation. What’s missing in all of this is using cholinergic antagonists to pharmacologically challenge efferent-mediated afferent excitation.

Since several studies have suggested the EVS plays some role in governing afferent sensitivity and timing as well as gain and plasticity of the vestibulo-ocular reflex (VOR)^31–35^, specific information regarding underlying synaptic mechanisms would be instructive in efforts to understand how EVS activation impacts normal vestibular physiology. To pharmacologically characterize mammalian EVS actions, we decided to use mice where future access to transgenic models and established pharmacological tools would provide additional insight into mammalian EVS function. In this study, single-unit recordings were acquired from mouse vestibular afferents in response to electrical stimulation of efferent neurons before and after application of cholinergic antagonists. The goals were to characterize responses of mouse vestibular afferents to EVS stimulation and identify if cholinergic mechanisms were involved.

## Results

### Afferent responses to midline EVS stimulation

**Figure 1:**
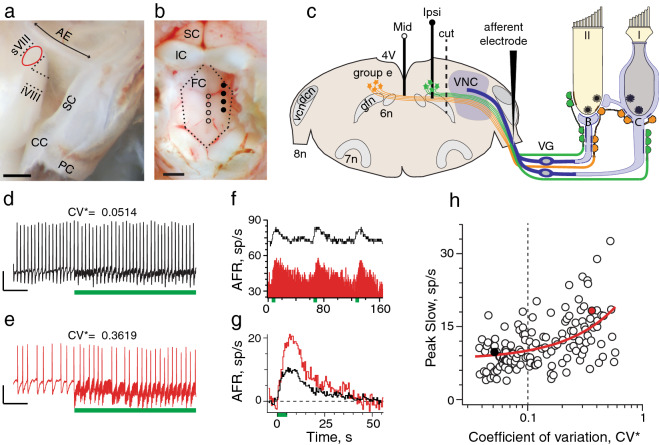
Electrical stimulation in the brainstem slowly excites vestibular afferents. (**a**) Right bony labyrinth w/dashed lines outlining positions of superior (sVIII) and inferior (iVIII) divisions of cranial nerve VIII (8n) dorsally obscured by the brainstem (i.e., cochlear nuclei). CC, common crus; SC, PC superior and posterior canal. Afferent recordings were confined to sVIII (red dashed oval), mediocaudal to the arcuate eminence (AE). (**b**) EVS electrode placement in floor of fourth ventricle: Circles—open, midline; filled, ipsilateral. FC, IC, SC: facial, inferior, and superior colliculus. (**c**) Coronal brainstem section showing EVS nuclei (i.e. group e) and projections to peripheral hair cells and afferents. In vestibular neuroepithelium, primary vestibular afferents (blue), innervating type I and/or type II hair cells, collect in 8n and project to the vestibular nuclear complex (VNC). In the brainstem, contralateral efferent neurons (orange) cross the midline to join ipsilateral efferents (green) with both exiting 8n. Efferent neurons pass through the vestibular ganglion (VG) without synapsing on cell bodies for primary vestibular afferents^8,86^. Upon reaching the neuroepithelium, EVS neurons produce many varicosities innervating type II hair cells and their bouton afferents (B), as well as calyx-bearing afferents (C) innervating type I hair cells. Key: 4V, 4th ventricle; 6n, abducens nucleus; 7n, facial nucleus; dcn, vcn, dorsal and ventral cochlear nuclei; gfn, genu of facial nerve. (**d,e**) Regularly (black; 2 mV, 100 ms) and irregularly-discharging (red; 0.5 mV, 100 ms) afferent showing onset of midline brainstem stimulation (333 shocks/s, green bar), respectively. (**f**) Corresponding continuous histograms showing changes in afferent firing rates (AFR) during three successive midline stimulation trials (333/s for 5 s delivered every 60 s, green boxes). (**g**) Corresponding average response histograms after background subtraction (n = 7 trials/unit). (**h**) Mean peak slow excitation, averaged from a 1-s block at t = 6–7 s, is plotted versus CV* for 144 units (n: 65 regular, 79 irregular). Vertical dashed line separates regular from irregular afferents. Filled color circles correspond to units shown in panel **g**. Red line; linear fit of the data. Scale bars: a = 1 mm; b = 0.5 mm. Binning in panels (**f)** and (**g)** is 500 ms.

We characterized extracellular spike responses of vestibular afferents during EVS stimulation to evaluate if efferent-mediated afferent responses in mice were similar to other mammals^2,12,13^. In anesthetized C57BL/6 mice, we advanced glass borosilicate microelectrodes into the superior division of cranial nerve VIII while stimulating efferent neurons in the dorsal brainstem with a platinum-iridium electrode array (Fig. 1a–c). Using a normalized coefficient of variation (CV*) to describe discharge regularity^36–38^, spike activity in 264 afferents from 106 animals were classified as either regularly (CV* < 0.1, n = 99) or irregularly-discharging (CV* > 0.1, n = 165) (Fig. 1d,e). Consistent with previous mouse studies^37–39^, mean discharge rates were higher in regular afferents (69.9 ± 1.8 vs. 35.6 ± 1.7 spikes/s; p < 0.0001).

Midline brainstem stimulation, predicted to recruit contralateral and bilateral EVS neurons (Fig. 1c), excited regular and irregular afferents (Fig. 1f–h). Unlike earlier studies where both efferent-mediated fast and slow excitation were typically observed^2,12,13^, midline stimulation in mice primarily elicited slow excitation in regular and irregular afferents. The resulting excitation was characterized as slow given time-to-peak, post-stimulus duration, and lack of abrupt rate changes during stimulus onset (Fig. 1f,g). Peak slow excitation, measured at t = 6–7 s, was significantly larger in irregular afferents (13.2 ± 0.6 spikes/s, n = 79 vs. 9.2 ± 0.6 spikes/s, n = 65, p < 0.0001, Mann–Whitney) (Fig. 1f–h) whereas duration was significantly longer in regular afferents (43.5 ± 1.2 vs. 33.8 ± 1.2 s, p < 0.0001, unpaired t-test). Despite a significant two-fold difference in mean slow excitation from selected subsets of regular and irregular afferents (9.7 ± 0.3 vs. 18.4 ± 1.4 spikes/s; n = 15, p < 0.0001, unpaired t-test), rise time constants, derived from exponential fits of the first 8 s of activation in each group, were indistinguishable (2.37 ± 0.20 vs. 2.39 ± 0.17 s, p = 0.9250, unpaired t-test), suggesting they were produced by similar mechanisms.

**Figure 2:**
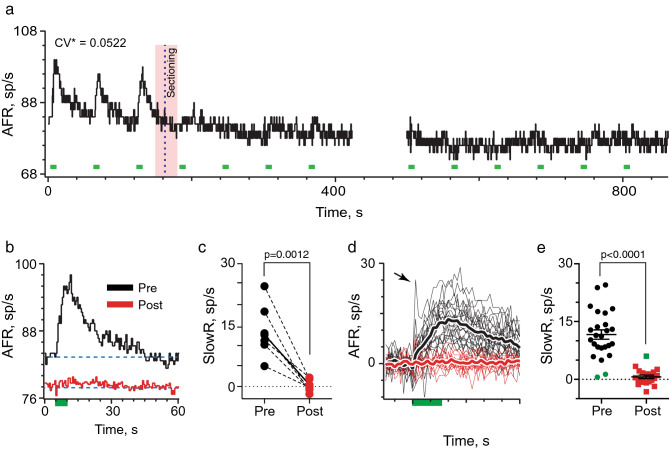
Electrical activation of vestibular efferents in the brainstem accounts for slow excitation of vestibular afferents. (**a**) Continuous response histogram from a regular afferent shows changes in afferent firing rate (AFR) during midline efferent stimulation (green bars, 333/s for 5 s every 60 s) before and after severing EVS neurons. Red box and dashed line indicate timing of a rostrocaudal cut in the brainstem using a small myringotomy blade mounted on a micromanipulator. Gap in trace at t = 425 s represents a 1-min interval between adjacent trials in the same unit. (**b**) Corresponding average response histograms from the same afferent in panel (**a)** were generated separately for 3 and 10 shock trains delivered before and after sectioning, respectively. (**c**) Mean peak slow excitation values, averaged from a 1-s block at t = 6–7 s, are plotted for seven afferents from seven animals before and after sectioning. Solid line depicts values from example in Panel (**b)**. Indicated p value from comparisons made using a paired t test. (**d**) Forty-five individual afferent response histograms to efferent shock trains (green bar, 333/s for 5 s) delivered before (black, n = 25) or after (red, n = 20) sectioning. AFR for each unit is plotted as a displacement from background. Thick traces represent average response histograms for each population. Arrow points to prominent efferent-mediated fast excitation in one unit. (**e**) Mean peak slow excitation values, averaged from a 1-s block at t = 6–7 s, are plotted for the 45 afferents in panel (**d)**. Population mean ± SEM are indicated by black bars. Solid green circles show pre-section units without efferent-mediated response while solid green square shows post-section afferent with a residual efferent-mediated response. Indicated p value from comparisons made using an unpaired t test. Binning in panels (**a**,**b**,**d)** is 500 ms.

To confirm that direct stimulation of EVS neurons drives slow excitation, we asked whether severing EVS neurons would abolish afferent responses to brainstem stimulation (see “cut”, Fig. 1c). This gold-standard approach, which eliminates action potential conduction along efferent axons, has confirmed roles for EVS stimulation in other mammals^2,13^. Consistent with EVS activation in the brainstem, afferent excitation during midline stimulation was abruptly eliminated after making a rostrocaudal cut between stimulating and recording electrodes (Fig. 2). Continuous recordings from the same vestibular afferent, before and after the cut, demonstrated that slow excitation was immediately eliminated following sectioning (Fig. 2a). The first three shock trains elicited slow afferent excitation averaging ~ 13 spikes/s (Fig. 2b). At t = 160 s when the brainstem cut is made, however, subsequent shock trains fail to produce excitation (Fig. 2a,b). In seven units, the mean slow excitation of 13.6 ± 2.4 spikes/s observed before sectioning was significantly different than the 0.2 ± 0.5 spikes/s observed after sectioning (Fig. 2c). The mean amplitude of post-sectional afferent responses was not significantly different from zero.

Effectiveness of this procedure in abolishing afferent responses to midline stimulation was also evaluated by comparing numbers of afferents excited by midline stimulation before and after sectioning. A total of 45 afferent recordings (25 pre-, 20 post-sectioning) were obtained in nine animals. Response histograms and population means are plotted in Fig. 2d. Sectioning eliminated nearly all afferent excitation associated with midline stimulation. Before sectioning, mean peak afferent excitation measured at t = 6-7 s was 11.6 ± 1.2 spikes/s, which was significantly different from the mean excitation of 0.7 ± 0.5 spikes/s obtained after the cut (Fig. 2e). Two afferents failed to respond before sectioning (green circles), while only one afferent exhibited a small excitation after sectioning (green square) suggesting some efferent fibres remained intact. Collectively, the sectioning data are consistent with the assertion that electrical stimulation in the mouse brainstem activates EVS neurons to excite vestibular afferents.

Spontaneous activity in vestibular efferents is documented in frog, fish and mammals^40–44^. Furthermore, recent data^45^ has suggested spontaneous activity in mouse EVS neurons augments vestibular afferent discharge, perhaps through continuous activation of the same peripheral mechanisms driving efferent-mediated slow excitation in our study. To assess whether EVS neurons were active in our preparation, we asked if the same rostrocaudal sectioning altered afferent baseline discharge. We reasoned that any tonic EVS influences on vestibular afferent activity should also be revealed by sectioning efferent axons and disrupting EVS signals from reaching the vestibular periphery. However, significant changes in afferent baseline activity were not seen after sectioning (Fig. 2a). In seven units held through sectioning, the mean afferent discharge rate before the cut was 46.7 ± 12.8 spikes/s versus 45.5 ± 12.8, 44.0 ± 12.9, and 43.2 ± 12.9 spikes/s, taken 10, 30, and 60 s after the cut, respectively. There were no statistically-significant differences between mean discharge rates as determined by one-way ANOVA (F(1.989, 11.93) = 2.562, p = 0.1188). There is a gradual 5–6 spikes/s decrease over time in the unit shown in Fig. 2a, but this decrease is in play before sectioning. Similar decreases are seen in recordings without sectioning and are likely attributed to changes in afferent excitability following initial electrode placement and/or residual slow excitation from subsequent EVS stimuli in preceding trials. To remove potential influence of prior brainstem stimulation on afferent activity, we performed sectioning experiments in five animals without stimulating EVS neurons. Here, mean baseline rate recorded before sectioning was 31.4 ± 11.2 spikes/s while mean baseline rates of 30.7 ± 11.6, 30.2 ± 11.6, and 30.5 ± 11.6 spikes/s were measured at 10, 30 and 60 s after sectioning, respectively. Again, differences between mean discharge rates were not statistically significant as determined using one-way ANOVA (F(1.963, 9.814) = 2.080, p = 0.1769). These observations suggest, at least in our preparation, that spontaneous EVS activity, if present, contributes little to elevating afferent discharge.

### Afferent responses to ipsilateral EVS stimulation

**Figure 3:**
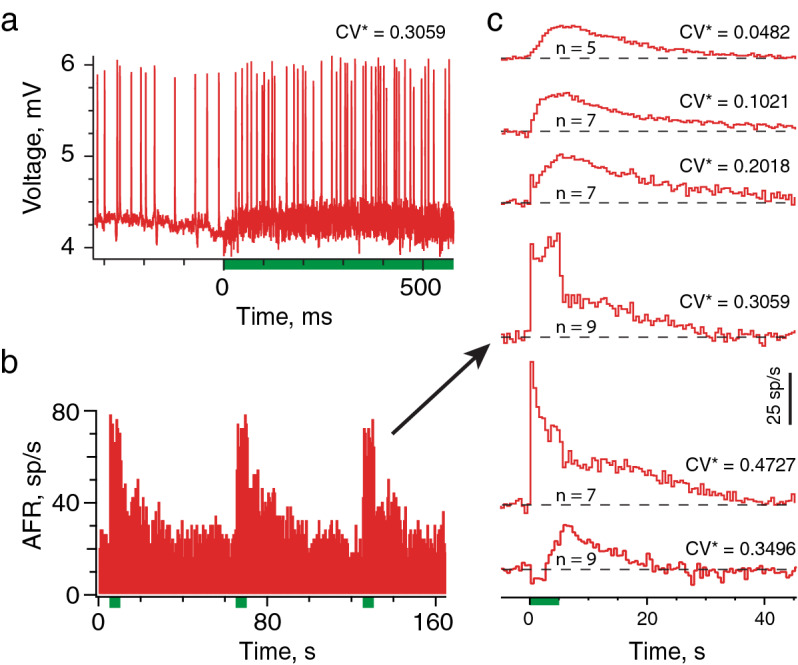
Ipsilateral efferent stimulation elicits fast and slow excitation. (**a**) Irregularly-discharging afferent showing the onset of ipsilateral brainstem stimulation (333 shocks/s, green bar). (**b**) Corresponding continuous histogram showing changes in afferent firing rates during three successive ipsilateral stimulation trials (333/s for 5 s delivered every 60 s, green boxes). Average response histogram shown to the right of arrow. (**c**) Average response histograms to ipsilateral efferent stimulation (background subtracted) in five units with different CV* values. Histograms are plotted as a displacement from background (dashed line). Number of trials used to construct mean histograms indicated in each trace. Binning in panels (**b,c)** is 500 ms.

Only a few large and fast excitatory responses were seen in irregular afferents with midline stimulation. An example is shown in Fig. 2d where the fast-excitatory peak is seen within the first 500 ms of the stimulus (arrow) occurring seconds before peak slow excitation. Scarcity of fast excitatory responses in mouse vestibular afferents during midline stimulation was unexpected given their prevalence in other mammals with similar EVS stimulation paradigms^2,12,13^. However, anatomical data in gerbils suggests that activation of ipsilateral efferent neurons are necessary to fully engage irregular afferents^8^. As midline stimulation only activates bilateral and contralateral EVS neurons (Fig. 1c), such anatomy, if present in mice, might account for our observations. Alternatively, recruitment of both contralateral and ipsilateral efferent neurons during stimulation may be necessary for larger responses in irregularly-discharging afferents, simply as a function of providing additional synaptic input. To test these ideas, we characterized afferent responses to efferent stimulation after placing stimulating electrodes, ~ 600–800 µm off the midline (Fig. 1b,c), corresponding to the location of ipsilateral EVS neurons^5,43,46,47^. Ipsilateral stimulation should recruit both contralateral and ipsilateral EVS neurons innervating end organs on one side. Like midline stimulation, efferent-mediated slow excitation of vestibular afferents was also seen during ipsilateral stimulation and was the predominant form of efferent-mediated excitation in regularly-discharging afferents at CV* ≤ 0.1 (Fig. 3). However, in many irregular afferents, ipsilateral stimulation also triggered a pronounced fast excitation in addition to the slow excitation (Fig. 3a,b). Efferent-mediated fast excitation was often visible for the entire stimulus and could exceed rates of 50 spike/s in some units (Fig. 3b,c). Efferent-mediated fast excitation without slow excitation was occasionally seen in irregularly-discharging afferents.

It is also worth noting that efferent-mediated inhibition, although infrequent, was seen with ipsilateral stimulation (Fig. 3c, bottom trace). Peak efferent-mediated inhibition, averaging − 13.8 ± 3.9 spikes/s and ranging from − 5.1 to − 27.8 spikes/s, was seen in 5 afferents (3 irregular, 2 regular). In three units, including the example in Fig. 3c, efferent-mediated inhibition was followed by post-inhibitory excitation, similar to efferent-mediated afferent responses in frog, turtle, and monkey^2,48,49^. Efferent-mediated inhibitory responses were not further characterized given their infrequent occurrence. We instead focused our efforts on efferent-mediated fast and slow excitation.

**Figure 4:**
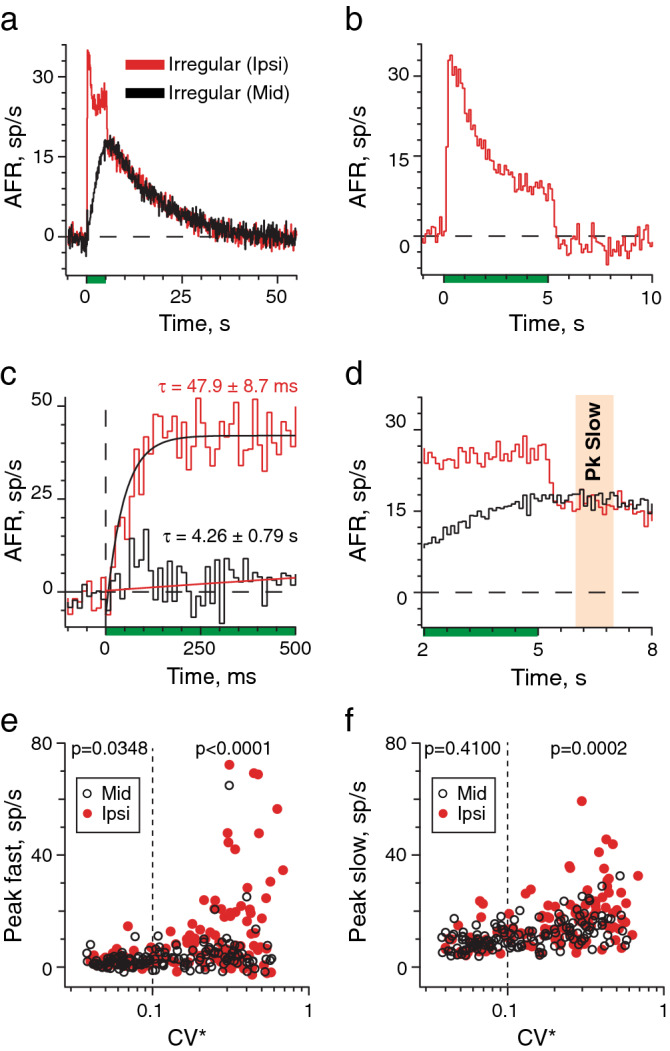
Efferent-mediated fast and slow excitation may be distinguished based on response activation and duration. (**a**) Ensemble mean response histograms (100 ms bins) were generated for irregular afferents during ipsilateral (red, 18 units, 108 trials) or midline efferent stimulation (black, 18 units, 74 trials). (**b**) Time course of efferent-mediated fast excitation is shown after subtracting the midline ensemble histogram from the ipsilateral ensemble histogram in panel (**a)**. (**c**) Ensemble mean response histograms from panel (**a)** were replotted using a smaller 12.5-ms bin size. The first 500-ms of the ensemble histograms are shown. Rise times constants were derived from exponential fits. (**d**) Expansion of panel (**a)** demonstrates that estimates of slow excitation at t = 6–7 s (orange bar) exclude contributions from fast excitation. (**e**) Mean efferent-mediated fast excitation for ipsilateral and midline stimulation are plotted against CV*. (**f**) Mean efferent-mediated slow excitation for ipsilateral and midline stimulation are plotted against CV*. Total number of afferents for midline and ipsilateral stimulation are 144 (n = 65 regular, 79 irregular) and 120 (n = 34 regular, 86 irregular), respectively. Vertical dashed line separates regular from irregular afferents. Indicated p values in panels (**e,f)** are from comparisons of response amplitudes in regular or irregular afferents with midline vs. ipsilateral stimulation using Mann Whitney.

To further characterize each form of efferent-mediated excitation, we selected 18 irregular afferents showing efferent-mediated fast and slow excitation with ipsilateral stimulation and compared those with 18 irregular afferents showing only efferent-mediated slow excitation with midline stimulation. Superimposition of ensemble average histograms for each stimulus location highlighted the time course of efferent-mediated fast excitation (Fig. 4a). The difference histogram, after subtracting midline from ipsilateral, revealed mean fast excitation decays by ~ 70% during the stimulus and quickly returns to baseline following stimulus termination (Fig. 4b). Exponential fits of each peak response reveal the rise time constant for fast excitation is nearly 90-fold faster than slow excitation (Fig. 4c). As such, the mean amplitude of slow excitation during the first 500-ms constitutes only ~ 8% of the fast response. Conversely, fast excitation is completed by ~ 300-ms after stimulus termination and does not contribute to peak slow excitation measurements at t = 6–7 s (Fig. 4d). Therefore, relatively uncontaminated estimates of peak fast and slow excitation were tabulated from histogram response segments at t = 0–0.5 s and t = 6–7 s, respectively (Figs. 4c–f). In irregularly-discharging afferents, large fast responses typically required ipsilateral EVS stimulation (Fig. 4e). Only seven instances of efferent-mediated fast excitatory responses > 10 spikes/s were seen with midline stimulation versus 36 seen with ipsilateral stimulation. While mean efferent-mediated slow excitation in regular afferents with midline stimulation were not significantly different from those obtained with ipsilateral stimulation, mean efferent-mediated fast excitatory responses in regular afferents as well as both fast and slow excitatory responses in irregular afferents were significantly larger with ipsilateral stimulation (Fig. 4e, f).

### Pharmacology of EVS-mediated afferent excitation

**Figure 5:**
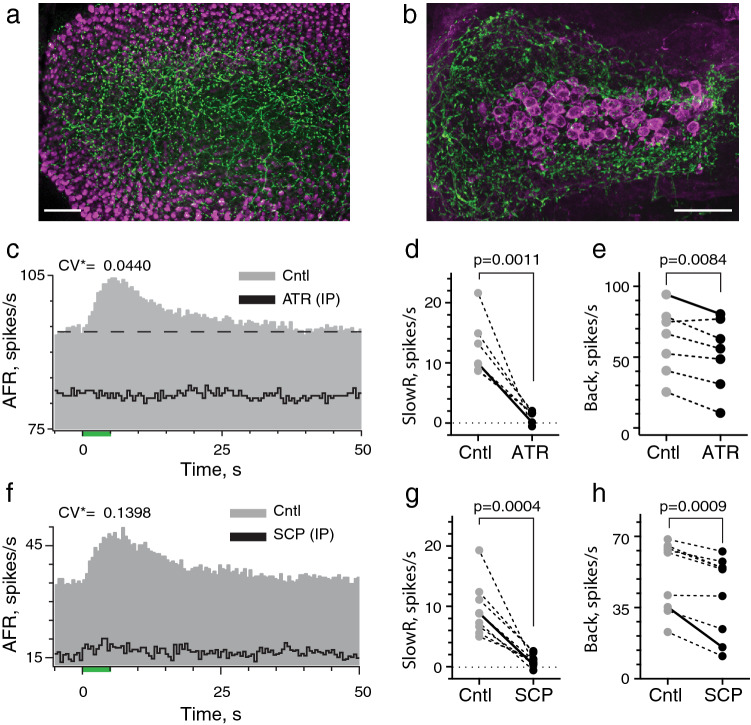
The muscarinic ACh receptor antagonists, atropine and scopolamine, block efferent-mediated slow excitation in mouse vestibular afferents. (**a,b**) Whole-mount immunofluorescence of the mouse saccular macula and canal hemicrista (dorsal view) showing that ChAT- positive EVS neurons (green) innervate the full extent of the neuroepithelium. Hair cells (**a**) and calyx afferents (**b**) are counterstained with antibodies against calretinin (magenta). Scale Bars: 50 µm. (**c**) Average response histograms showing the effects of midline efferent stimulation in a regular afferent before (Cntl, gray) and after IP administration of 0.5 mg/kg atropine (ATR, black). Histograms in each condition were tabulated from 7 efferent shock trains. (**d,e**) Mean peak slow excitation (SlowR) and background discharge rates (Back) for seven individual units during control and atropine conditions (EVS stimulation: midline, n = 4; ipsilateral, n = 3). (**f**) Average response histograms showing the effects of midline efferent stimulation in an irregular afferent before (Cntl, gray) and after IP administration of 0.5 mg/kg scopolamine (SCP, black). Histograms for control and SCP conditions were tabulated from 6 and 8 efferent shock trains, respectively. Binning in panels (**c,f)** is 500 ms. (**g,h**) Mean peak slow excitation (SlowR) and background discharge rates (Back) for nine individual units during control and scopolamine conditions (EVS stimulation: midline, n = 6; ipsilateral, n = 3). Example histograms shown in panel (**c,f)** appear as solid lines in panels (**d**,**e,g**,**h)**, respectively. In population data from both atropine (**d,e**) and scopolamine (**g,h**), the range and mean doses were 0.2–2 mg/kg and 0.75 mg/kg, respectively. Indicated p values from comparisons made using a paired t test.

Differences in kinetics of EVS-mediated fast and slow afferent excitation suggest they utilize different postsynaptic efferent mechanisms. This is further supported by observations that efferent-mediated slow and fast excitation can be evoked independently. It is well established that the mammalian EVS is cholinergic^1,2,5,11,43,47,50^. In C57BL/6 mice, cholinergic efferent varicosities, labelled with antibodies to choline acetyltransferase (ChAT), heavily populate the entire otolithic macula and canal crista suggesting ACh is available to drive both forms of efferent-mediated excitation in mouse vestibular afferents (Fig. 5a,b). Furthermore, like turtle^17,18^, differences in response kinetics between slow and fast excitation could be explained by activation of muscarinic (mAChR) and nicotinic (nAChR) ACh receptors, respectively. To determine if cholinergic mechanisms were involved, we obtained efferent-mediated responses in individual vestibular afferents before and after administration of selective cholinergic antagonists.

We first concentrated on efferent-mediated slow excitation which we speculated was driven by mAChRs given their role in similar responses in turtle^18^. Here, the mAChR antagonists atropine and scopolamine were administered via intraperitoneal (IP) injections after first obtaining control recordings during EVS stimulation. In a regular unit (Fig. 5c), IP atropine (0.5 mg/kg) completely blocked efferent-mediated slow excitation. When tested against regular and irregular afferents in seven animals, atropine blocked 90% of the mean efferent-mediated slow excitation (12.4 ± 1.8 vs. 1.2 ± 0.4 spikes/s) (Fig. 5d). IP atropine also reduced afferent background discharge, seen as downward shifting of the average response histogram during blockade of efferent-mediated slow excitation (Fig. 5c, black trace). In seven units, IP atropine significantly decreased the mean afferent discharge rate from 61.8 ± 9.0 to 52.4 ± 9.4 spikes/s (Fig. 5e). IP atropine’s mean time-to-block was 15.4 ± 4.8 min. This considerable length of time was influenced primarily by two units requiring > 30 min to block. In these two animals, we speculate atropine was given subcutaneous, rather than IP, which accounts for slower delivery. Exclusion of these units reduced the block time to 8.4 ± 1.7 min which is consistent with IP delivery times of other drugs in our experiments. Comparable effects on efferent-mediated slow excitation were seen with IP scopolamine (Fig. 5f). In regular and irregular afferents from nine animals, scopolamine significantly blocked over 90% of the mean efferent-mediated slow excitation (9.4 ± 1.5 vs. 0.9 ± 0.3 spikes/s) and reduced afferent background discharge from 50.7 ± 5.8 to 41.7 ± 6.5 spikes/s (Fig. 5g,h). IP Scopolamine’s mean time-to-block was 6.3 ± 1.3 min. Under similar conditions, IP saline failed to significantly affect efferent-mediated slow excitation (10.4 ± 2.1 vs. 10.7 ± 2.4 spikes/s, p = 0.4638) or background discharge (79.4 ± 11.8 vs. 77.2 ± 11.8 spikes/s, p = 0.4785) in seven afferents (data not shown). Blockade by atropine and scopolamine suggests efferent-mediated slow excitation of mouse vestibular afferents requires the activation of mAChRs.

The effects of mAChR antagonists on baseline afferent activity were somewhat surprising. While sectioning data suggested spontaneous activity in efferent neurons was not contributing to afferent baseline discharge, spontaneous ACh release from efferent terminals is seen in several hair cell preparations^51–53^ and, if present in our preparation, could tonically activate mAChRs and elevate afferent baseline discharge. Under these conditions, mAChR antagonists in addition to blocking efferent-mediated slow excitation would also reduce afferent firing rates. Alternatively, atropine and scopolamine, when delivered systemically, have access to peripheral and central mAChRs in tissues beyond the ear. In the 6–8 min required for mAChR antagonists to block efferent-mediated slow excitation, additional off-target effects may impact the excitability of vestibular afferents (e.g. cardiovascular) and decrease afferent baseline discharge. To preferentially deliver mAChR antagonists quickly to peripheral EVS synapses, we developed an intrabulla (IB) approach to introduce drug solutions to the middle ear for round window entry into the perilymph. Similar approaches have been used to deliver numerous substances to the mouse’s inner ear^54–56^.

**Figure 6:**
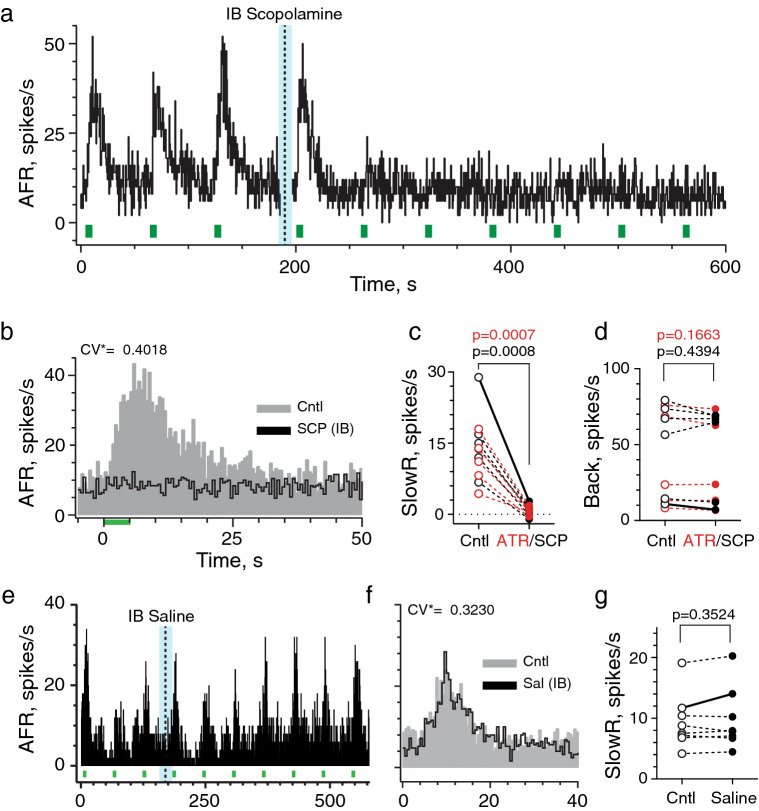
Intrabulla application of atropine and scopolamine rapidly blocks efferent-mediated slow excitation in mouse vestibular afferents. (**a**) Continuous response histogram from an irregular afferent shows changes in afferent firing rate (AFR) during midline efferent stimulation (green bars, 333/s for 5 s every 60 s) before and after intrabulla delivery (IB) of scopolamine (30 µl at 0.5 mM). Scopolamine was delivered to the middle ear in a 10-s window between trials (blue box and dashed line). (**b**) Corresponding average response histograms from the same afferent in panel (**a)** were generated separately for 5 efferent shock trains delivered before and after IB scopolamine (SCP). Binning = 500 ms. (**c**) Values of mean peak slow excitation during control (Cntl) and atropine (red, ATR) or scopolamine (black, SCP) conditions. (**d**) Values of mean background discharge during control (Cntl) and atropine (red, ATR) or scopolamine (black, SCP) conditions. Example histograms shown in panel (**b)** appear as solid lines in panels (**c,d)**. (**e**) Continuous response histogram from an irregular afferent shows changes in afferent firing rate (AFR) during midline efferent stimulation (green bars, 333/s for 5 s every 60 s) before and after intrabulla delivery of saline (30 µl). Saline was delivered to the middle ear over a 10-s window between trials (blue box and dashed line). (**f**) Corresponding average response histograms from the same afferent in panel **e** were generated separately for efferent shock trains delivered before (Cntl) and 3–4 min after IB saline (Sal). The y-axis labels are the same as panel (**e**). Binning = 500 ms. (**g**) Values of mean peak slow excitation during control (gray, Cntl) and after IB saline (black, ATR). Example histograms shown in panel (**f)** appear as solid lines in panels (**g)**. Indicated p values from comparisons made in panels (**c**,**d**,**g)** using a paired t test.

Provided mAChR antagonists quickly and selectively enter the inner ear, IB administration should discriminate whether reduction in background activity is attributed to blockade of tonic mAChR activation or other off-target systemic effects. In Fig. 6a, a continuous rate histogram reveals the effects of IB scopolamine (30 µl @ 0.5 mM) on an irregularly-discharging afferent with large efferent-mediated slow excitation. The afferent’s response to repeated efferent shock trains (333 shocks/s for 5 s every 60 s) are shown before and after delivery of scopolamine to the middle ear. Three points can be made here. One, like IP scopolamine, IB scopolamine completely blocked efferent-mediated slow excitation (Fig. 6a,b). Two, blockade of slow excitation with IB scopolamine was faster than IP consistent with its relative proximity to the inner ear. The afferent responds to the first EVS shock train immediately after IB scopolamine and response amplitude/duration are comparable to preceding trials during the control period (Fig. 6a). However, efferent-mediated slow excitation is blocked during the next EVS shock train 60 s later, and stays blocked for the remainder of the recording. Third, despite sensitivity of this afferent to EVS stimulation, IB scopolamine had little effect on background discharge (Fig. 6a,b) suggesting that decreases in afferent firing seen with IP scopolamine are not associated with mAChR blockade in the ear.

IB scopolamine was tested in regular and irregular afferents in six animals where it blocked > 95% of mean efferent-mediated slow excitation (15.6 ± 3.0 vs. 0.8 ± 0.5 spikes/s) while no significant changes in mean afferent background discharge (50.3 ± 12.3 to 48.2 ± 12.2 spikes/s) were observed (black symbols; Fig. 6c,d). Similar observations were made for mean efferent-mediated slow excitation (12.8 ± 1.5 vs. 0.5 ± 0.4 spikes/s; 96% block) and mean background discharge (37.9 ± 14.3 vs. 36.0 ± 13.5 spikes/s) in five units when challenged with IB atropine (red symbols; Fig. 6c,d). The mean time to block for IB scopolamine (1.6 ± 0.3 min) and IB atropine (2.8 ± 0.5 min) were 3–4 times faster than mean block times observed for either drug given IP. Under similar conditions, however, IB saline had no effect on efferent-mediated slow excitation in an irregular unit (Fig. 6e,f). Like IP saline, IB saline failed to significantly alter efferent-mediated slow excitation (9.5 ± 1.6 vs. 9.8 ± 1.8 spikes/s; Fig. 6g) or background discharge (56.2 ± 10.8 vs. 56.9 ± 11.5 spikes/s; p = 0.5771; data not shown) in eight afferents. Collectively, these data demonstrate for the first time that efferent-mediated slow excitation of mammalian vestibular afferents utilizes mAChR activation during EVS stimulation.

**Figure 7:**
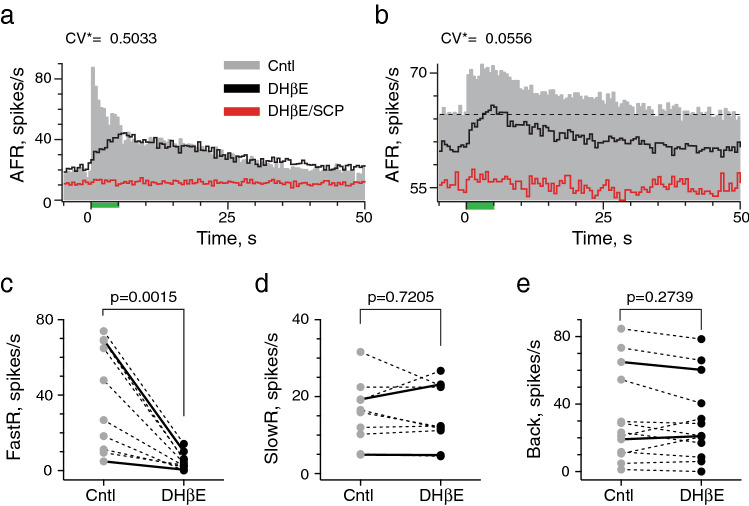
Efferent-mediated fast excitation is selectively blocked by the nicotinic ACh receptor antagonist DHβE. (**a,b**) Average response histograms from an irregularly- and regularly-discharging afferent during ipsilateral stimulation, respectively, before (gray, Cntl) and after serial administration of DHβE (black, 1.4 mg/kg) and scopolamine (red, SCP, 1 mg/kg). Binning in panels (**a**,**b)** is 500 ms. (**c,d**) Values of mean peak fast (FastR) and slow excitation (SlowR) for ten units before and after DHβE. The range and mean doses were 1.4–5 mg/kg and 3.5 mg/kg, respectively. (**e**) Values of mean background discharge rates for 13 individual units before and after DHβE application. Examples shown in panel (**a**,**b)** appear as solid lines in panels (**c–e)**. Indicated p value from comparisons made using a paired t test.

The above observations establish a role for ACh in the generation of efferent-mediated slow excitation, but direct pharmacological evidence that ACh also gives rise to efferent-mediated fast excitation in mice or other mammals is lacking. Efferent-mediated fast excitation of vestibular afferents in turtle relies on activation of α4α6β2*nAChRs^16–18^. We sought to determine whether similar mechanisms exist in mice by characterizing efferent-mediated fast excitation of vestibular afferents before and after IP administration of dihydro-β-erythroidine (DHβE), an α4α6β2*-selective nAChR antagonist that blocks efferent-mediated fast excitation in turtle^16,17^. Efferent-mediated fast excitation in an irregular (Fig. 7a) and regular afferent (Fig. 7b) were identified as abrupt increases in afferent discharge at the beginning of the efferent shock train. In both units, DHβE (1.4 mg/kg, IP) blocked the fast excitation with no effect on efferent-mediated slow excitation (black histograms). That slow excitation in these units was mediated by mAChRs was confirmed following blockade with subsequent IP scopolamine. DHβE was tested in afferent recordings from thirteen animals including three afferents only exhibiting efferent-mediated fast excitation and three afferents only exhibiting efferent-mediated slow excitation. Collectively, DHβE significantly blocked near 90% of the mean efferent-mediated fast excitation (39.6 ± 8.9 vs. 4.8 ± 1.4 spikes/s, n = 10) without affecting mean efferent-mediated slow excitation (15.7 ± 2.6 vs. 15.2 ± 2.5 spikes/s, n = 10) (Fig. 7c,d). Unlike mAChR antagonists, IP DHβE had no systematic effect on afferent baseline activity (Fig. 7e). The mean baseline discharge rate in the thirteen units before DHβE application, 32.9 ± 7.6 spikes/s, was not significantly different from the mean discharge rate of 30.7 ± 6.7 spikes/s recorded after DHβE. DHβE’s time to block was 6.0 ± 0.7 min, comparable to delivery times associated with IP scopolamine and atropine. These data confirm that ACh release during EVS stimulation and subsequent nAChR activation drives efferent-mediated fast excitation in mouse vestibular afferents.

## Discussion

This is the first study to characterize responses of mouse vestibular afferents to EVS stimulation. Electrical stimulation of vestibular efferents elicited a fast and slow afferent excitation that varied as a function of afferent discharge regularity and site of efferent stimulation. Efferent-mediated slow excitation required 6–8 s to peak and post-stimulus durations of ≥ 30 s before returning to baseline, while efferent-mediated fast excitation peaked and decayed within hundreds of milliseconds from the start and termination of the efferent shock train. The amplitude and duration of efferent-mediated fast and slow excitation in mice overlap similar measurements in other species (Table 1). In mouse, efferent-mediated slow excitation routinely occurred in regular and irregular afferents with midline or ipsilateral EVS stimulation while efferent-mediated fast excitation was more frequently observed in irregular afferents when stimulating ipsilaterally. Efferent-mediated fast and slow excitation were larger in irregular afferents under both stimulation locations. Finally, pharmacological data show for the first time that responses of mammalian vestibular afferents to EVS stimulation are mediated by activation of distinct cholinergic mechanisms.

**Table 1:** Comparison of response metrics for efferent-mediated fast and slow excitation among different vertebrates.

Response metric	Mouse (this study)	Monkey^2^	Chinchilla^13^	Cat*^12^	Turtle*^17,18^
Fast Amp (spikes/s)	3–15 (0–72)	3.8–65.7	7.6–43.9	10–60	35–46
Slow Amp (spikes/s)	9.1–19 (3–43)	5.3–36.3	8.2–21.0	20–100	16–18
Fast Dur (s)	5–6	5–6	5–6	0.3–0.5	1.25–10
Slow Dur (s)	34–44	25–30	30–40	70–85	145

The range of mean response amplitudes (Amp) and durations (Dur) are provided for several mammalian species and turtle. Efferent-mediated response metrics from regular and irregular afferents have been pooled to generate the range except for cat and turtle where data were only available for irregular afferents (*). Number in parentheses for mouse are the ranges of response amplitudes taken from individual units. Amp and Dur measurements were taken from average response histograms generated with different efferents stimuli—Mouse, squirrel monkey, and chinchilla: 1–12 shock trains (333 shocks/s for 5 s) repeated every 60–75 s; Cat: 64 shock trains with each train (200 shock/s for 400 ms) presented at 1.5 s intervals; Turtle, 10–25 shock trains (200 shocks/s for 100 ms) or a single shock train (100 shock/s for 10 s). Longer response durations in cat and turtle are attributed to the length of the efferent stimulus. References for each animal are shown as superscripts.

Efferent-mediated fast excitation was blocked by DHβE while efferent-mediated slow excitation was antagonized by atropine and scopolamine, indicating roles for nAChRs and mAChRs, respectively. Similarities in response properties suggest that fast and slow excitation in other mammals likely use comparable mechanisms^2,12,13^. Sensitivity of fast excitation to DHβE and slow excitation to atropine mirrors pharmacological data in turtle implicating α4α6β2*-nAChRs and odd-numbered mAChRs in efferent-mediated fast and slow excitation of calyx-bearing afferents^17,18^. These nAChR subunits and mAChRs are expressed in the mammalian vestibular periphery^19–25^, but further pharmacological characterization is required to specify nAChR and mAChR subtypes.

Large efferent-mediated fast excitatory responses in squirrel monkey and chinchilla were seen in irregular afferents with midline and ipsilateral EVS stimulation^2,13^. These observations indicate both contralateral and ipsilateral efferents target irregular afferents consistent with anatomical data in chinchilla showing both groups innervating neuroepithelial regions where irregular afferents reside^7^. In mice, however, efferent-mediated fast excitation was larger and more frequent in irregular units with ipsilateral stimulation suggesting some irregular afferents may receive ipsilateral, but not contralateral, EVS innervation. This configuration is supported by anatomical data in gerbil suggesting contralateral efferents selectively innervate regular afferents while ipsilateral efferents target irregular afferents^8^. Data in mice, however, are not entirely consistent with gerbil anatomical details as midline stimulation excited both regular and irregular afferents, and efferent-mediated fast excitation was seen occasionally with midline stimulation. A more plausible explanation is ipsilateral and contralateral efferents overlap in their innervation, such that dual recruitment during ipsilateral stimulation provides additional efferent synaptic input to fully activate fast afferent excitation. This explanation is consistent with mean efferent-mediated fast and slow excitation being significantly larger in irregular afferents with ipsilateral stimulation.

Like mouse, efferent-mediated inhibitory responses, although infrequent, were reported for squirrel monkey^2^. Similar responses in frog and turtle are mediated through α9nAChRs-SK activation that hyperpolarizes type II hair cells to reduce transmitter release and inhibit afferent discharge^16,17,57–59^. Recently, patch clamp data has demonstrated the α9nAChR-SK mechanism is widely present in mouse type II hair cells^26,27^, which begs the question why efferent-mediated afferent inhibition is not commonly observed? In fish and turtle^17,60^, one explanation is inhibitory influences on afferent discharge during EVS stimulation are often masked by competing efferent-mediated excitatory mechanisms. While we speculate efferent-mediated inhibition of mouse vestibular afferents is attributed to α9nAChRs-SK, pharmacological confirmation is needed. Targeting efferent-mediated excitation first with selective nAChR and mAChR blockers will be helpful in isolating the underlying α9nAChR-mediated afferent inhibition and probing EVS function. To this end, studies in α9KO mice have suggested the mammalian EVS and α9nAChRs play critical roles in several vestibular-related phenomena^33–35,61^.

Spontaneous activity in EVS neurons occurs in several species^40–44^. Were such activity to release ACh and activate mAChRs driving efferent-mediated slow excitation, it could elevate afferent background discharge. Support for this idea comes from a study suggesting photothermal stimulation in anesthetized mice inhibits EVS neurons and reduces afferent activity^45^. However, in our preparation, we were unable to demonstrate a role for spontaneous efferent activity as neither severing EVS neurons nor IB mAChR blockade with scopolamine or atropine had significant effects on afferent background discharge. Spontaneous efferent activity is sensitive to the choice of general anaesthetic agents. The aforementioned studies performing direct efferent recordings were done in preparations with minimal to no general anaesthetic on board^40–44^. Furthermore, different general anaesthetics are associated with varying decreases in efferent activity in vestibular, auditory, and lateral line systems^62–65^. Differences in spontaneous EVS activity here and Raghu et al.^45^ could be related to use of urethane versus ketamine anaesthesia. Further studies regarding anaesthetic effects on mammalian EVS function are warranted.

Scopolamine is widely used to alleviate motion sickness in mammals, including humans and mice, which likely occurs due to a sensory mismatch of peripheral stimuli (i.e. vestibular, vision, and proprioception) with the expected central representation of motion^66–70^. Efferent-mediated fast excitation and inhibition reduce the sensitivity of vestibular afferents to vestibular stimuli^2,48^, while efferent-mediated slow excitation enhances afferent gain^18,30^, suggesting they play opposing roles in governing afferent responsiveness. These mechanisms may be key to modifying afferent gain during adaptation of vestibular reflexes or asymmetries in vestibular input^33–35^. Excessive mAChR activation during efferent-mediated slow excitation and the associated increase in afferent sensitivity could contribute to the mismatch. In addition to targeting mAChRs in central vestibular circuitry^66,68,71^, our data show that scopolamine also blocks mAChRs in the vestibular periphery. These peripheral actions, along with central actions as an anti-emetic and sedative, may account for scopolamine’s effectiveness in the clinical setting^72–74^. Downstream mechanisms for mAChR activation likely include KCNQ potassium channel closure^18,28,30^ suggesting that titrating KCNQ channel activity might represent another strategy for alleviating motion sickness. Going forward, these pharmacological agents, as well as those targeting nAChRs, should be useful in evaluation of mammalian EVS function and directing subsequent EVS studies in transgenic animals.

## Materials and methods

### Mice

All mouse procedures, in accordance with NIH’s Guide for the Care and Use of Laboratory Animals, were approved by University Committee for Animal Resources at the University of Rochester Medical Centre (URMC), and the authors complied with the ARRIVE guidelines. Experiments were conducted in C57BL/6 mice of either sex, weighing 19–30 g, and aged 49–120 days. Animals, obtained from Charles River Laboratories or Jackson Laboratory, were housed in one-way rooms with a standard 12-h light:dark cycle with free access to food and water.

### Surgical procedures

Mice were deeply anesthetized with IP urethane/xylazine (1.6 g/kg/20 mg/kg). After tracheostomy, PTFE tubing (1.5-cm long, 1-mm diameter) was inserted into the trachea and secured with surgical thread ligatures^75^. Mice were rotated to the prone position and the tracheal cannula was connected to a ventilator maintained at a rate of 100 bpm (model 683, Harvard Apparatus). Body temperature (36.9–37.8 °C) was maintained with a homiothermic monitoring system and heart rate was monitored using a 3-lead EKG. Mice were secured using a bite plate and non-puncturing ear bars in a stereotaxic frame (Stoelting). Dorsal skin overlying the skull was removed and the posterior neck muscles were detached with a round knife to expose cranial sutures and 1st cervical vertebrae. Occipital/intraparietal bones were removed with micro-rongeurs to expose the cerebellum and inferior colliculi. The right transverse venous sinus was occasionally cauterized and divided when extending the craniotomy rostrally. The vermis was aspirated using a 5Fr Frazier suction exposing the floor of the fourth ventricle. Finally, the right cerebellar hemisphere, flocculus, and parafloculus were aspirated using a #22 suction to expose the arcuate eminence, common crus, and access to the right superior and inferior division of cranial nerve VIII (Fig. 1a). Bleeding was minimized using small cotton pledgets.

### Afferent recordings

Borosilicate microelectrodes (BF150-86-10, Sutter Instrument) with impedances of 40–120 MΩ were filled with 3 M KCl, loaded onto a Burleigh Inchworm drive, and connected to a preamplifier headstage (Biomedical Engineering, Thornwood, NY). After identifying cranial nerve VIII by gently retracting the brainstem, microelectrodes were advanced into the superior division to record extracellular spike activity from spontaneously-discharging vestibular afferents. Afferents were classified according to discharge regularity by computing interspike interval (ISI) statistics from a 5-s segment of background activity. An interval coefficient of variation (CV) was derived from the mean ISI and its standard deviation, (CV = SD_ISI_/MEAN_ISI_). We used the power function, CV = a(CV*)^b^, to normalize CV (CV*) to a 15-ms interval using previously reported coefficients in chinchilla^36^. Although mouse normalization coefficients are also available^37^, chinchilla values were selected to permit CV* determination for afferents with discharge rates of 90–125 spikes/s^36,38^.

### Efferent stimulation

A linear electrode array of four Teflon coated platinum-iridium wires was lowered into the floor of the fourth ventricle^76^. For midline stimulation, the array was placed between and caudal to the facial colliculi (See Fig. 1b) while for ipsilateral stimulation, the array was moved rightward ~ 600–800 µm from the midline. Five seconds of background activity were acquired before the first efferent shock train was delivered. To facilitate comparisons with other mammalian preparations^2,13^, electrical stimuli consisted of 5-s trains of 100–150 μs constant current shocks delivered at 333 shocks/s between any two array wires. Shock amplitude was adjusted to determine threshold (T, 20–50 µA) and maxima (75–300 µA) that elicited afferent responses without antidromic activation of primary afferents. Inter-trial intervals of 60–75 s were used for afferent discharge to return to near pre-stimulus values before delivering the next shock train.

Afferent responses to EVS stimulation in mice are typically represented as an average response histogram (ARH) constructed from 3–12 individual stimulation trials under each experimental condition. Shock train start was set at t = 0 and spike times were specified for each trial from 5 s before each EVS shock train to 50–65 s after each train. Afferent responses to multiple efferent shock trains were also expressed as continuous rate histograms to visualize single runs of multiple shock trains and reveal the serial effects of a particular treatment. Most histograms were segmented in 500 ms bins and total number of spikes per bin was transformed to a discharge rate after dividing by bin width. The mean peak amplitude of efferent-mediated fast excitation was tabulated from the first 500-ms segment of the ARH starting at t = 0 s. The mean peak amplitude of efferent-mediated slow excitation was computed from a 1-s segment at t = 6–7 s, a region overlapping the natural peak of efferent-mediated slow excitation but uncontaminated by efferent-mediated fast excitation. Reported response amplitudes include a subtraction of mean background discharge rates taken from the 5-s prestimulus interval (T = − 5 to 0) of the ARH. Duration of efferent-mediated slow excitation was determined by identifying the time point where the mean prestimulus discharge rate intersects the exponential fit of the response decay back to baseline.

### Brainstem sectioning

A spear-tipped myringotomy knife (377122, Beaver-Visitec), mounted to a 3-axis micromanipulator, was lowered in brainstem, to make a rostrocaudal section in the brainstem between the stimulating array and recording electrode to sever efferent projections to peripheral end organs (See Fig. 1c). The midline efferent electrode remained intact during the sectioning. Afferent responses to midline efferent stimulation were recorded before and after the sectioning.

### Data acquisition

Efferent stimuli delivery and data acquisition were managed using in-house Spike2 scripts on a PC with a micro1401 interface (Cambridge Electronic Design). Afferent signals were low-pass filtered (1 kHz, four-pole Bessel; Wavetek) and sampled at 10 kHz. Timing of efferent shock trains was controlled from a digital-output port routed to a stimulus isolator (WPI). Spike2 data files were exported as general text files and processed with custom macros in IgorPro 6.36 (WaveMetrics). We minimized stimulation artefacts offline by subtracting a computed average single shock artefact from each shock stimulus in the raw data.

### Drug administration

To probe efferent receptor mechanisms, afferent responses to efferent stimulation were obtained before, during, and after pharmacological agents were administered by either intraperitoneal (IP) injection or direct delivery into the right middle ear space using an intrabulla (IB) approach. Both approaches have demonstrated that drugs applied by either method reaches the inner ear space^54–56,77^. The IB approach required making a small incision behind the right ear and retracting the underlying muscles to expose the posterior bulla. A small hole was then made in the thin portion of the otic bulla with a 30-G needle and a customized plastic syringe pulled to a ~ 200-µm tip was inserted and sealed with cyanoacrylate glue. IP injection of *d*-tubocurarine (dTC, 0.625 mg/kg) was used to suppress muscle contractions that often accompany brainstem stimulation^76^. Experimental drugs included the mAChR antagonists atropine (ATR, 0.2–2 mg/kg,) and scopolamine (SCP, 0.2–2 mg/kg) as well as the nAChR antagonist dihydro-β-erythroidine (DHβE, 1.44–5 mg/kg). Atropine and DHβE were selected given their effects against efferent-mediated afferent excitation in turtle^16–18^, while interest in scopolamine was related to its common clinical use in treating motion sickness^66,67^. As inner ear drug entry is governed by the same principles underlying drug access into the CNS^78,79^, the above dosing ranges were selected from previous in vivo mouse studies where the administration of atropine^80–82^, scopolamine^68,70,83^, or DHβE^84,85^ was used to target central cholinergic mechanisms. IB delivery of atropine or scopolamine typically used a 30 µL volume at 0.3–0.5 mM. Source of drugs used in this study: Atropine and scopolamine (URMC pharmacy or Sigma), DHβE (Tocris), and dTC (Sigma).

### Immunohistochemistry

Mice were anesthetized with IP ketamine/xylazine (100/10 mg/kg) and perfused with 4% (w/v) PFA freshly prepared in phosphate-buffered saline (PBS, 0.9% NaCl in 0.1 M PB). As described previously^11^, vestibular labyrinths were extracted from the temporal bone of three C57BL/6 mice and immunohistochemistry was performed on microdissected vestibular organs with antibodies to ChAT (1:100, Millipore) and calretinin (1:1000; Millipore). Anti-ChAT was used to label vestibular efferent fibres and varicosities while anti-calretinin was used primarily to counterstain hair cells in the otolithic macula and calyx afferents in the crista central zone^11^. Whole mount projections were acquired either with a Zeiss Axio Imager motorized upright multifluorescent microscope fitted with an Apotome slider system (Zeiss Imaging Systems, Oberkochen, Germany) or Olympus FV1000 Laser Scanning Confocal microscope. Images were postprocessed using Axiovision, Olympus FV1000, and/or Adobe Photoshop CS6 (Adobe Systems, San Jose, CA) software to adjust contrast and brightness within the linear range.

### Statistical procedures

Differences in the amplitudes of fast/slow efferent-mediated responses between regular/irregular afferents or between ipsilateral/midline stimulation were evaluated using Mann Whitney. Afferent response amplitudes before/after brainstem sectioning were evaluated using a paired or unpaired t test. Effects of sectioning on baseline discharge rates as well as drug effects on efferent-mediated afferent responses and background discharge were assessed using a paired t test. One-sample t-test was used to evaluate if means differed from zero. One-way ANOVAs were performed for comparisons of background rates before/after sectioning. All statistical analyses were done in Graph Pad-Prism (GraphPad). Values, expressed as means ± SEM, and outcome parameters including p values are reported in the text and figures.

## Data Availability

The bulk of the data generated or analysed for this study are included in this published article. Datasets generated during the current study are available from the corresponding author on reasonable request.
